# Impact of Sprouting under Potassium Nitrate Priming on Nitrogen Assimilation and Bioactivity of Three *Medicago* Species

**DOI:** 10.3390/plants11010071

**Published:** 2021-12-27

**Authors:** Ahlem Zrig, Ahmed Saleh, Foued Hamouda, Mohammad K. Okla, Wahidah H. Al-Qahtani, Yasmeen A. Alwasel, Abdulrahman Al-Hashimi, Momtaz Y. Hegab, Abdelrahim H. A. Hassan, Hamada AbdElgawad

**Affiliations:** 1 Faculty of Sciences of Gabès-City Erriadh, Zrig, Gabes 6072, Tunisia; 2 Department of Botany, Faculty of Science, Cairo University, Giza 12613, Egypt; asaleh@sci.edu.eg; 3 Research Unit in Enterprise and Decisions, Higher Institute of Management, Road Jilani Habib, Gabes 6002, Tunisia; foha2001@gmail.com; 4 Botany and Microbiology Department, College of Science, King Saud University, Riyadh 11451, Saudi Arabia; okla103@yahoo.com (M.K.O.); yasmeen@ksu.edu.sa (Y.A.A.); aalhashimi@ksu.edu.sa (A.A.-H.); 5 Department of Food Sciences and Nutrition, College of Food and Agriculture Sciences, King Saud University, Riyadh 11451, Saudi Arabia; wahida@ksu.edu.sa; 6 Resarch Institute of Medicinal and Aromatic Plants, Beni-Suef University, Beni-Suef 62511, Egypt; momtaz.hegab@science.bsu.edu.eg; 7 Department of Food Safety and Technology, Faculty of Veterinary Medicine, Beni-Suef University, Beni-Suef 62511, Egypt; abdelrahim@vet.bsu.edu.eg; 8 Integrated Molecular Plant Physiology Research, Department of Biology, University of Antwerp, 2020 Antwerp, Belgium; hamada.abdelgawad@uantwerpen.be

**Keywords:** sprouts, *Medicago* species, priming, KNO_3_, nitrogen assimilation, bioactivity

## Abstract

Edible sprouts are rich in flavonoids and other polyphenols, as well as proteins, minerals, and vitamins. Increasing sprout consumption necessitates improving their quality, palatability, and bioactivity. The purpose of this study was to test how KNO_3_ priming affects the sprouting process species on three *Medicago* species (*Medicago indicus*, *Medicago interexta*, and *Medicago polymorpha*) and their nutritional values. Targeted species of *Medicago* were primed with KNO_3_, and the levels of different primary and secondary metabolites were determined. KNO_3_ induced biomass accumulation in the sprouts of the three species, accompanied by an increased content of total mineral nutrients, pigments, vitamins, and essential amino acids. Besides, our results showed that KNO_3_ enhanced the activity of nitrate reductase (NR), glutamate dehydrogenase (GDH), and glutamine synthetase (GS) enzymes, which are involved in the nitrogen metabolism and GOGAT cycle, which, in turn, increase the nitrogen and protein production. KNO_3_ treatment improved the bioactive compound activities of *Medicago* sprouts by increasing total phenolic and flavonoid contents and enhancing the antioxidant and antidiabetic activities. Furthermore, species-specific responses toward KNO_3_ priming were noticeable, where *Medicago interexta* showed the highest antioxidant and antidiabetic activities, followed by *Medicago polymorpha*. Overall, this study sheds the light on the physiological and biochemical bases of growth, metabolism, and tissue quality improvement impact of KNO_3_ on *Medicago* sprouts.

## 1. Introduction

One of the natural processing methods for increasing the nutritional value and health qualities of foods is the sprouting of seeds. This approach has been employed in Eastern countries for a long time [1]. Since the sprouts are eaten so early in their growth cycle, their nutrient content remains very high [2]. Sprouts are excellent sources of protein, vitamins, and minerals, as well as essential nutrients for promoting health, such as glucosinolates and phenolic components [3]. Besides, the phytochemicals, enzymes, and amino acid contents in sprouts are very beneficial for human health [2]. On the other hand, some public concerns stem from the danger of bacterial contamination (e.g., *E. coli*, *Salmonella enterica*, and *Vibrio cholerae*) of sprouts because they are typically prepared at home and served as salad ingredients without any thermal or other sanitary treatment [4]. Different seeds may be sprouted for human consumption, including legume seeds (bean, pea, lentil, soybean), grains (rye, wheat, barley, oats), and, more recently, seeds of certain vegetables (alfalfa, radish). Due to consumer demand for minimally processed, additive-free, more sustainable, nutritional, and balanced foods, sprouting of seeds is gaining popularity in all countries [4].

*Medicago* genus is one of the first plants to be cultivated. It literally means “Father of All Foods.” It is also known as “the queen of forages” since it has been employed in the food business as a cheap source of protein, particularly as a fodder plant. *Medicago* sprouts contain high amounts of vitamins A and C, coumestrol, liquiritigenin, isoliquiritigenin, loliolide, and saponins [4]. Furthermore, the high content of protein, and bioactive compounds found in both the aerial and root sections of the lucerne plant, have sparked a lot of interest in its cultivation [5]. Because of their high level of bioactive phytochemicals, such as phenolic components, saponins (hederagenin and soyasapogenol), and essential amino acids, lucerne formulations have antifungal, antibacterial, insecticidal, and nematicidal characteristics (valine, leucine, threonine, and lysine). In addition, *Medicago* species can be used as an effective functional ingredient in the dietary prevention and treatment of several metabolic conditions, particularly the metabolic syndrome, due to its high content of proteins, minerals, isoflavones, and other substances with estrogenic activity, anti-inflammatory properties, and antioxidant activity [6]. In this regard, *Medicago* sprout seed sprouting is one of the processing ways for increasing the nutritional content of this leguminous. Human consumption of lucerne is generally modest; however, there has been a growing interest in using this plant as green salad sprouts, pills, or drinks for their influence on blood cholesterol in various nations [7].

Potassium (K) is an essential plant nutrient that regulates a variety of metabolic activities, including protein synthesis and glucose metabolism [8]. Exogenous application of K fertilizers, such as potassium nitrate (KNO_3_), monopotassium phosphate (KH_2_PO_4_), and potassium sulfate (K_2_SO_4_), has been shown to improve nutrient uptake, plant growth, and photosynthesis, as well as mitigate the abiotic stress [9]. The same as K, nitrogen (N) is an essential element that has a direct impact on plant development and physiological processes [10]. N assimilation is an important physiological process that influences plant productivity and quality [10]. Nitrate and ammonium are the most important sources of nitrogen for plant growth and development, with nitrate being more important [11]. Nitrate reductase (NR) and nitrite reductase (NIR) are enzymes that catalyze the conversion of nitrate to nitrite and then nitrite to ammonium [12]. In a cyclic manner, glutamine synthetase (GS, EC 6.3.1.2) and glutamate synthase (EC 1.4.7.1) or glutamine 2-oxoglutarate aminotransferase (GOGAT, EC 1.4.7.1) absorb ammonium to create distinct amino acids [13]. Amino acids, non-pharmacological and non-toxic universal nutrients, are the primary building blocks for protein synthesis in cells [14]. Finally, proteins are classified, modified, transported, and stored to become constituent parts of plant life [15].

Seed priming is a pre-sowing technique for controlling seedling growth by modulating pre-germination metabolic activities prior to radicle emergence, which improves germination rate and plant output in general [16]. Hydro-priming (soaking in water), osmo-priming (soaking in osmotic solutions, such as polyethylene glycol, sodium, and potassium salts), solid matrix priming, biopriming (coating with bacteria, such as *Pseudomonas aureofaciens*, AB254), and treatment with plant growth regulators (PGRs), combined with priming medium, are all examples of priming methods [16]. Priming with KNO_3_ improves seed germination, mineral composition, proline, amylase, and protein pattern [17]. It improves seedling establishment and vigor and has a remarkable role in the pre-sowing accomplishment of germination phases [18]. However, the detailed metabolic events induced by KNO_3_ during the sprouting process are not quite understood. Therefore, the purpose of this study was to assess the impact of KNO_3_ priming on growth, N metabolism, tissue quality, and biological values in the sprouts of three species of *Medicago*.

## 2. Materials and Methods

### 2.1. Plant Material and Growth Conditions

Seeds of *Medicago* (*Medicago indicus*, *Medicago interexta*, and *Medicago polymorpha*) were collected from the Agricultural Research Center (Giza, Egypt), where they were collected during filed trips to different locations in Egypt (Giza and Ismailia). Before being stored in distilled water overnight, the seeds were rinsed in distilled water and submerged in 5 g L^−1^ sodium hypochlorite for 1 h. Two hundred seeds were clustered into a group soaked in distilled water (control), and the second group was soaked in a KNO_3_ solution of 25 mM, for 16 h. The applied growth promoting concentration of KNO_3_ was selected according to a pilot experiment, where five concentrations (0 and 10, 15, 25, 50 mM) were tested. Each species seeds were evenly placed on 10 vermiculite-lined trays, which were irrigated every two days with Milli-Q water, and each tray received 150 mL of aquaponic water. The experiment was conducted in a growth cabinet under controlled conditions (25 °C, 16 h light/8 h dark cycle, and PAR of 400 µmol m^−2^ s^−1^ and relative humidity of 60% per day). After ten days, the fresh mass of each sprout was measured and then stored at −80 °C for further biochemical analyses. Experiments were repeated 4 times, and 20 plants that were pooled from each tray and treated (biological replicate) were used for each measurement.

### 2.2. Analysis of Mineral Contents

Two hundred milligrams of each KNO_3_-primed *Medicago* sprout was processed in an oven with an HNO_3_/H_2_O solution (5:1 *v*/*v*) to determine macro and micro-elements. The concentrations of macro-minerals and trace elements at 25 °C were measured using inductively coupled plasma mass spectrometry (ICP-MS, Finnigan Element XR, and Scientific, Bremen, Germany), with nitric acid in one percent employed as standards [19]. Tandard solution of multielement was used for calibration solution preparation at concentrations form 0 to 50 ppm in 2% HNO_3_. Nitric acid was used as blank. *Medicago* sprout samples were vaporized, atomized, and ionized inside the chamber of the plasma. The limits of detection (LODs) and quantification (LOQs) values ranged from 0.0002 to 0.01 ug kg^−1^ and 0.004 to 0.3 µg kg^−1^, respectively.

### 2.3. Determination of Leaf Pigments 

The frozen *Medicago* sprout samples (0.5 g) were homogenized in acetone for 1 min at 7000 rpm using a MagNALyser (Roche, Vilvoorde, Belgium), and then centrifuged for 20 min at 14,000× *g* at 4 °C [20]. Acrodisc GHP filter (0.45 µm/13 mm) was used to filter the supernatant. The solution was then evaluated by HPLC (Shimadzu, SPDM10Avp, Japan, Tokyo) at reversed-phase and at 4 °C [19]. Pigment and carotenoid separation was carried out on a silica-based C18 column (Waters, Spherisorb, 5 m ODS1, 4.6 250 mm) with two different solvents: (A) acetonitrile: methanol: water in the ratio of 81:9:10, and (B) methanol: ethyl acetate in the ratio of 68:32. A diode-array detector (Shimadzu SPDM10Avp) was used to analyze the extraction of chlorophyll a and b, beta-carotene, and xanthophylls at four distinct wavelengths (420, 440, 462, and 660 nm), respectively.

### 2.4. Determination of Amino Acids

*Medicago* sprouts (300 mg fresh weight (FW)) samples were extracted in methanol [19]. GC/MS (Hewlett Packard, Palo Alto, CA, USA) analysis was carried out, and samples were separated on a HP-5 MS column. Two hundred milligrams of FW sprout leaves were homogenized in 80% aqueous ethanol and centrifuged at 22,000× *g* for 25 min to measure the amino acids. The supernatant was evaporated, and the precipitates were resuspended in chloroform. The pellet was re-dissolved in chloroform and was filtered (0.2-μm Millipore microfilters). Amino acids levels were measured by Waters Acquity UPLC-tqd system at 37 °C, low pressure, and mobile phase acetonitrile/water ration 60/40, with a measurement at 254 nm. The result was expressed in mg/g dry weight of the sample.

### 2.5. Determination of Polyphenols and Flavonoid Contents

One hundred milligrams of frozen sprouts were homogenized in 1 mL of 80% ethanol (*v*/*v*) to extract polyphenols and flavonoids [21]. The supernatant was utilized to determine the total phenolic and flavonoid contents after centrifugation at 4 °C for 20 min. A Folin–Ciocalteu test, with gallic acid as a standard, was used to assess phenolic content. The modified aluminum chloride colorimetric method was used to quantify flavonoid concentration, utilizing quercetin as a standard [22].

### 2.6. Determination of Vitamin Contents 

Using UV and/or fluorescence detectors, the amounts of ascorbate, tocopherols, thiamine, and riboflavin in sprouts were measured [19]. For thiamine, and riboflavin extraction, 250 mg of sample were extracted in 0.1 N HCl for 30 min [23]. Samples were separate through a 5 μm C18 Luna Phenomenex stainless steel column (250 × 4.6 mm i.d.). The mobile phase (methanol:sodium acetate (40:60 *v*/*v*) was used, and the fluorometric detection was performed at 453 nm and 580 nm, for riboflavin, and 366 nm and 453 nm, for thiamine. At 4 °C, ascorbate (vitamin C) was extracted in 1 mL of 6% (*w*/*v*) meta-phosphoric acid and separated using reversed-phase HPLC with a UV detector (100 mm 4.6 mm Polaris C18-A, 3 lm particle size; 40 °C, isocratic flow rate: 1 mL min1, elution buffer: 2 mM KCl, pH 2.5 with O-phosphoric acid). Tocopherol (vitamin E) was separated on a Particil Pac 5 m column (length 250 mm, i.d. 4.6 mm) and measured using HPLC (Shimadzu’s Hertogenbosch, normal phase conditions) and a fluorometric detector (excitation at 290 nm and emission at 330 nm). On a reverse-phase (C18) column, riboflavin and thiamine were separated (HPLC, methanol:water as mobile phase, and fluorescence as a detector).

### 2.7. Determination of Total Proteins

The protein was measured according to the Folin-Lowry. Two-tenths of a gram of frozen *Medicago* sprouts were homogenized in chloroform/methanol (2:1, *v*/*v*) solution and centrifuged for 15 min at 3000× *g* to measure the total proteins content [24].

### 2.8. Determination of N, Ammonium, and Nitrate Contents

The amount of nitrate in the water was determined using Cataldo et al.’s method [25]. Here, 0.1 mL filtrate and 0.4 mL 5% salicylic acid in concentrated H_2_SO_4_ made up the reaction mixture. After cooling at ambient temperature for 15 min, 9.5 mL 2 M NaOH was progressively added to elevate the pH above 12. The absorbance was measured at 410 nm when the solution was cooled to room temperature. Nitrate concentration was determined using a KNO_3_ calibration curve and represented in mg NO_3_-g^−1^ FW. The amount of ammonium in the sample was determined using the indophenol blue colorimetry method at 630 nm. The standard was ammonium chloride. Total N was measured using fine ground leaf dry samples (0.2 g) digested with H_2_SO_4_–H_2_O_2_ at 260 °C.

### 2.9. Determination of Antioxidant and Antidiabetic Activities

#### 2.9.1. Antioxidant Activity

Each *Medicago* sprout sample (0.1 g) was extracted in 80 percent ethanol and centrifuged for 20 min at 14,000 rpm [26]. The experiment was carried out in vitro using ferric reducing antioxidant power (FRAP) to determine antioxidant capabilities. In this case, we used 0.25 mL of FRAP reagent, mixing FeCl_3_ (20 mM) in acetate buffer (0.25 M, pH 3.6) at room temperature with 0.1 mL of diluted extract. For concentration calculation, calibration curve was performed using the standard Trolox (0.05–1 mM) as standard.

#### 2.9.2. Antidiabetic Activity

##### α-Amylase Inhibition Assay

The inhibition of pancreatic α-amylase inhibition was measured using *Medicago* sprout extract mixed with reaction solution starch (1 g/L) and phosphate buffer (pH 6.9). Then, 3 U/mL amylase enzyme was added to start the process [27]. After 10 min of incubation, 0.5 mL dinitro salicylic (DNS) reagent was added to terminate the reaction. The reaction mixture was heated to 100 °C for 10 min. Finally, the mixes received 0.5 mL of a 40 percent potassium sodium tartrate solution. At 540 nm, the absorbance was measured.

##### α-Glucosidase Inhibition Assay

The sprout hydroethanolic extract was combined with -glucosidase (2 U/mL) and incubated at 37 °C for 5 min to determine the inhibition of α-glucosidase [27]. After adding 1 mM para-nitrophenyl glucopyranoside dissolved in 50 mM phosphate buffer, the reaction buffer was incubated for 20 min at 37 °C (pH 6.8). A solution of sodium carbonate (1 M) was added to stop the process. The amount of para-nitrophenolate produced by para-nitrophenyl glucopyranoside was measured at 405 nm, and the inhibitory activity of -glucosidase was computed. The α-glucosi- dase inhibitory activity was expressed as percent inhibition and determined as follows: %inhibition = [(average A 405 control − average A 405 extractÞ/average A 405 control × 100].

#### 2.9.3. Glycemic Index GI

The GI was determined using an in vitro starch hydrolysis method [27]. The process begins with the incubation of *Medicago* sprouts in a reaction buffer of HCl-KCl buffer (pH 1.5) for one hour at 40 °C under shaking conditions with pepsin (100 mg/mL). The mixture was then diluted in phosphate buffer (pH 6.9) before being incubated at 37 °C with α-amylase. 1 mL of aliquots were collected every 30 min and boiled for 20 min to cease the activity of the amylase enzyme. To convert the remaining starch to glucose, 0.4 M sodium acetate buffer (pH 4.75) and 60 L amyloglucosidase were added. For 50 min, the reaction mixture was incubated at 60 °C. Approximately 0.6 mL aliquots were collected and incubated at 37 °C for 35 min with 1.2 mL glucose oxidase/peroxidase. The mixture’s absorbance was measured at 500 nm. The proportion of hydrolyzed starch at different times (0, 30, 60, 90, 120, and 180 min) was used to calculate the starch digestion rate. The area under the hydrolysis curve (AUC, 0–180 min) and hydrolysis were computed. The hydrolysis index was then computed by multiplying the difference between the AUC for a sample and the AUC for a standard by 100.

### 2.10. Statistical Analyses

The R statistics package was used to conduct the statistical analysis (Gplot, Agricola). All data were subjected to a one-way analysis of variance (ANOVA). As a post-hoc test for mean separations, Tukey’s Test (*p* = 0.05) was used. Each experiment was repeated at least three times (*n* = 3). The R software created hierarchical clustering using Heatmap (Pearson correlation). The COrplot package was used to do correlation analysis on all of the data.

## 3. Results 

### 3.1. Growth and Photosynthetic Pigments of Medicago Sprouts

**Figure 1:**
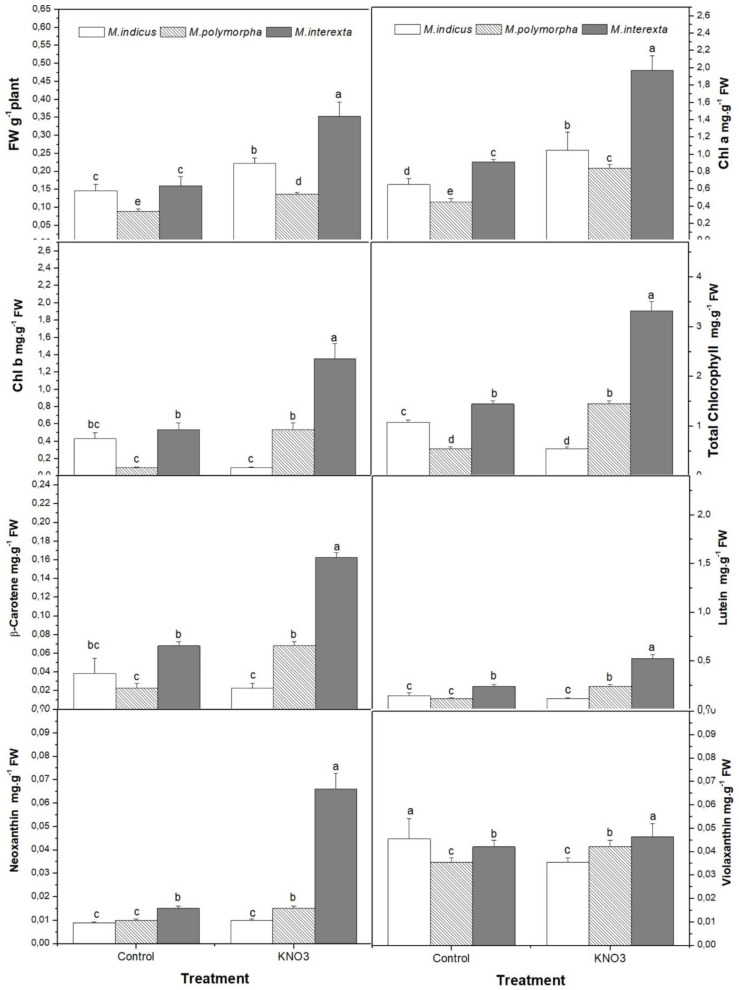
Effect of KNO_3_ priming on total fresh weight and leaf pigments contents of three species of *Medicago* sprouts. Values are represented by mean ± standard deviation of at least three independent replicates. Within the same species, different letters on the bars indicate significant differences at *p* < 0.05.

The fresh weight of sprouts showed a significant difference as a consequence of KNO_3_ priming. As shown in Figure 1, the sprout primed with KNO_3_ exhibited a significant increase in fresh weight (FW) in the three species, as compared to the control. Among the three species, the highest and most significant increase in FW was measured from *Medicago interexta* seeds priming with KNO_3_ by 53%, while the FW of both *Medicago indicus* and *Medicago polymorpha* primed with KNO_3_ increased only by 34% and 35%, respectively, as compared to control. In terms of the impact of KNO_3_ priming on photosynthetic pigments, there was a clear rising trend in *Medicago interexta* sprout leaf pigments, which showed increases in chlorophyll a (Chla), chlorophyll b (Chlb), and total chlorophyll of 53%, 60%, and 56%, respectively, as compared to control (Figure 1). Additionally, significant increases in Chla, Chlb, and total chlorophyll by 46%, 75%, and 56%, respectively, were recorded in *Medicago polymorpha*, as compared to control. Meanwhile, *Medicago indicus* sprouts showed an increase in chla by 38% and in total chlorophyll by 27%, while a slight increase was recorded in chlb. Furthermore, a significant amount of variation in carotenoid compounds in response to KNO_3_ priming was associated with differences among *Medicago* species. Indeed, the highest increase of β-carotene, lutein, and Neoxanthin by 54% 58%, and 70% was recorded in *Medicago interexta* sprouts (Figure 1). However, violaxanthin pigment was significantly increased in *Medicago polymorpha* and *Medicago indicus* by 36% and 35%, respectively, as compared to control.

### 3.2. Improvement of Nutritive Values: Mineral Content, Vitamins, and Antioxidant Activities

The Ca, K, Mg, Fe, Mn, and Zn concentrations, as well as Cu, were determined in dried *Medicago* sprouts. Regarding total nutrients analysis, the Ca, K, and Zn concentrations accounted for 24%, 22%, and 31%, respectively, on average in all species and treatment. However, Cu, Mg, Mn, and Fe were present in small amounts (Table 1). The mineral content of sprouts is very dependent on the sprouting conditions and species. The KNO_3_ priming increased the Ca, Cu, Fe, K, and P in the three species. Indeed, the greatest accumulation of K and P, by 2- and 5-fold, respectively, was observed in *Medicago interexta* sprouts. Likewise, KNO_3_ priming increased the content of Zn in *Medicago polymorpha* and *Medicago indicus* by 36% and 21%, respectively, as compared to control, while no significant change was observed in *Medicago interexta* sprouts. Furthermore, Mn and Mg content increased by 36% and 37% in *Medicago polymorpha*, and by 50% and 26% in *Medicago interexta.* In contrast, the Mn and Mg were not affected by KNO_3_ priming in *Medicago indicus.*

An analysis of vitamins was carried out for three species of *Medicago* sprouts (Table 2). The results revealed that Vit C presented the greatest accumulation by 41%, 32%, and 39% in *Medicago indicus*, *Medicago polymorpha*, and *Medicago interexta*, respectively (Table 1). KNO_3_ priming enhanced the Vit C, Vit E, and riboflavin content in all three species. Obviously, Vit C content was higher in *Medicago interexta* in comparison to other species. Likewise, both Vit E and riboflavin showed the highest accumulation by 46% and 39%, respectively, in *Medicago polymorpha* compared to control and other species. In contrast, a slight increase in thiamine was recorded in *Medicago indicus* in a response to KNO_3_ priming, and no significant change in *Medicago polymorpha.* While KNO_3_ priming enhanced the thiamine content in *Medicago interexta* by 2-fold compared to control conditions.

### 3.3. Amino Acid Metabolism 

HPLC analysis of amino acids in *Medicago* sprouts species revealed the presence of eighteen amino acids, with significantly different concentrations (Table 3). The highest value was recorded for glutamine in *Medicago interexta* (2.17 mg/g dry wt), followed by asparagine, glycine, phenylalanine, serine, proline, threonine, isoleucine, valine, leucine, lysine, tryptophane, cysteine, histidine, alanine, methionine, arginine, and tyrosine. KNO_3_ priming improved the accumulation of glutamine, serine, arginine, alanine, proline, histidine, valine, methionine, cystine, isoleucine, leucine, phenylalanine, tyrosine, lysine, threonine, and tryptophan in the three species. Indeed, the greatest accumulation was recorded in glutamine by 49% in *Medicago interexta* sprout. Likewise, Arginine showed the highest increase by 60% and 58% in *Medicago indicus* and *Medicago polymorpha*, respectively, compared to control. In contrast, the rest of amino acids were not significantly affected by KNO_3_ priming, depending on *Medicago* species. Compared to control sprouts, the N content was significantly affected by KNO_3_ in the three species. Unlike, the activities of nitrate reductase (NR), GDH, GOGAT, and GS were changed in response to KNO_3_ priming, depending on species (Table 3). Indeed, the highest increase of NR, GDH, GOGAT, and GS, by 26%, 32%, 32%, and 19%, respectively, was recorded in *Medicago interexta* sprouts. Furthermore, the protein content decreased in *Medicago indicus* and *Medicago polymorpha*, whereas it increased by 13% in *Medicago interexta*, compared to control.

### 3.4. Antioxidant and Antidiabetic Avtivities 

#### 3.4.1. Antioxidant Metabolites and Free Radical Scavenging Activity of *Medicago* Sprouts

**Figure 2:**
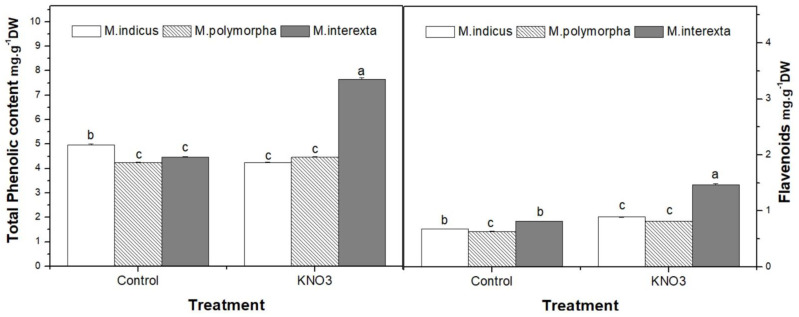
Effect of KNO_3_ priming on total phenolic and flavonoid contents of three species of *Medicago* sprouts. Values are represented by mean ± standard deviation of at least three independent replicates. Within the same species, different letters on the bars indicate significant differences at *p* < 0.05.

The present results revealed that the priming with KNO_3_ during germination has induced changes in phenolic compounds concentrations in *Medicago* sprouts (Figure 2). KNO_3_ priming significantly increased total phenolic and flavonoid contents in the three species.

**Figure 3:**
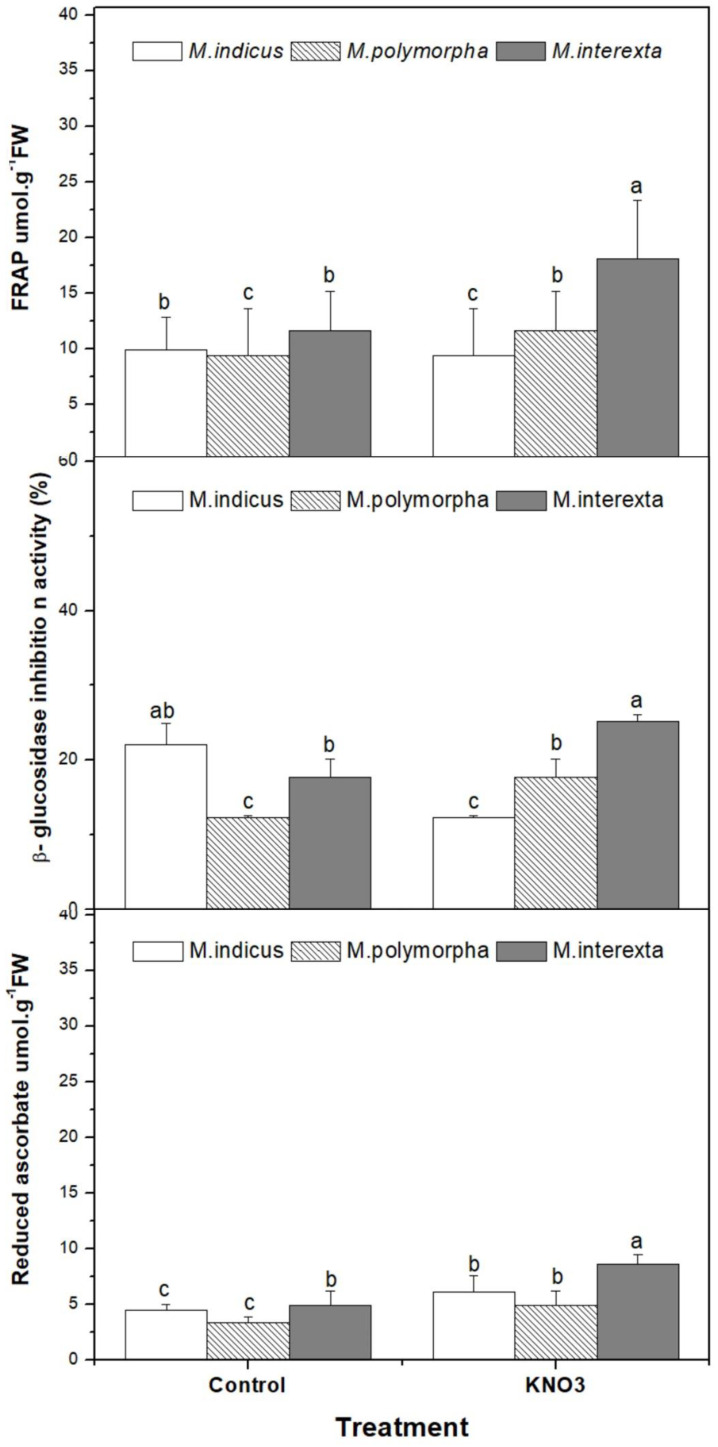
Effect of KNO_3_ priming on antioxidant activity of three species of *Medicago* sprouts. Values are represented by mean ± standard deviation of at least three independent replicates. Within the same species, different letters on the bars indicate significant differences at *p* < 0.05.

Besides, KNO_3_ priming enhanced the FRAP activities by 41%, 59%, and 35% for *Medicago indicus*, *Medicago polymorpha*, and *Medicago interexta*, respectively, in comparison to control (Figure 3). Furthermore, the reduced ascorbate and glutamine increased significantly (*p* < 0.05) in the three species as response to KNO_3_ priming, and the highest increase was recorded in *Medicago interexta* (Figure 3).

#### 3.4.2. Antidiabetic Activity

**Figure 4:**
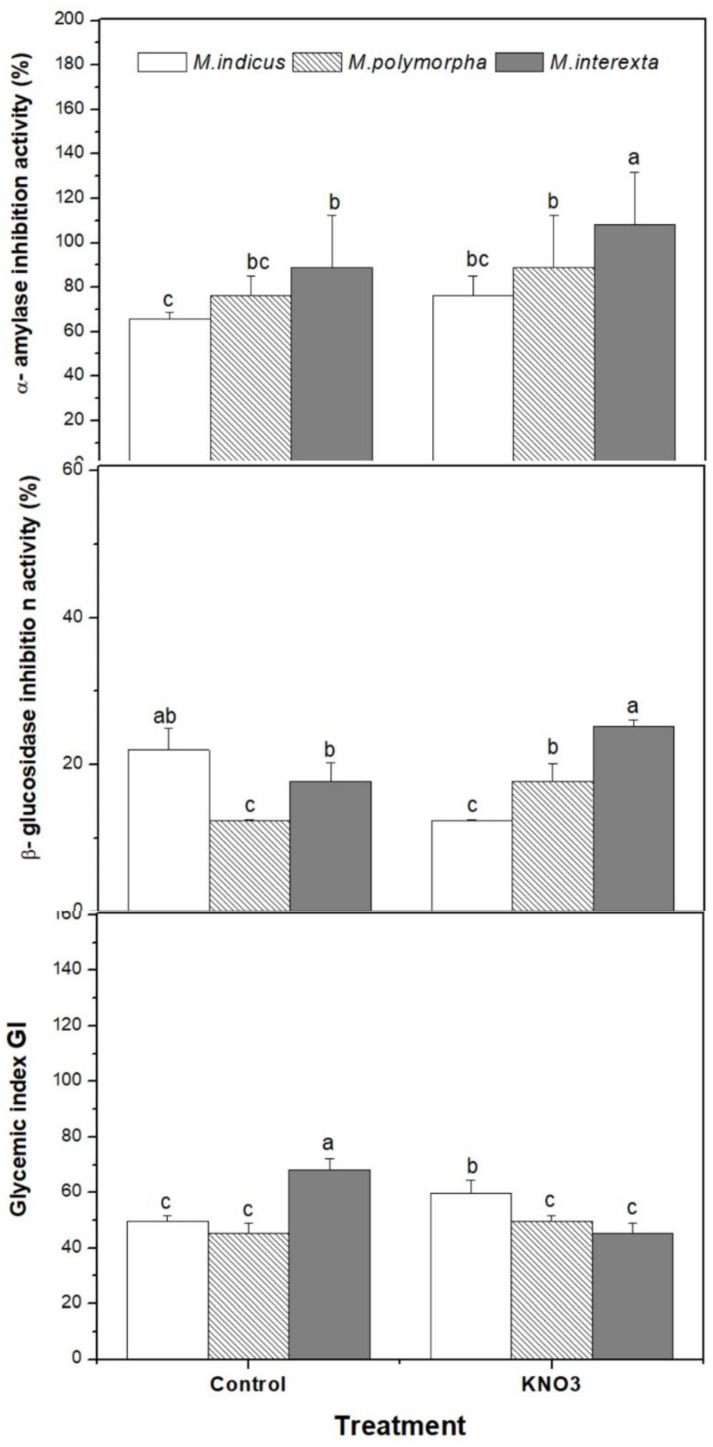
Effect of KNO_3_ priming on antidiabetic activity of three species of *Medicago* sprouts. Values are represented by mean ± standard deviation of at least three independent replicates. Within the same species, different letters on the bars indicate significant differences at *p* < 0.05.

As shown in Figure 4, each species of *Medicago* sprouts exhibited antidiabetic activity. Under control conditions, *Medicago interexta* had the best inhibitory effect on α-amylase, while *Medicago indicus* had the best inhibitory effects on α-glucosidase. KNO_3_ priming seemed to enhance the inhibition activity of α-amylase by 28% in *Medicago indicus*. Similarly, KNO_3_ priming enhanced the inhibition activity of α-glucosidase by 30%, 40%, and 29% for *Medicago indicus*, *Medicago polymorpha*, and *Medicago interexta*, respectively, compared to control. The current results also demonstrated that KNO_3_ priming caused a marked decrease in GI in *Medicago polymorpha* and *Medicago interexta* (lower than 70).

### 3.5. Principal Component Analysis (PCA)

**Figure 5:**
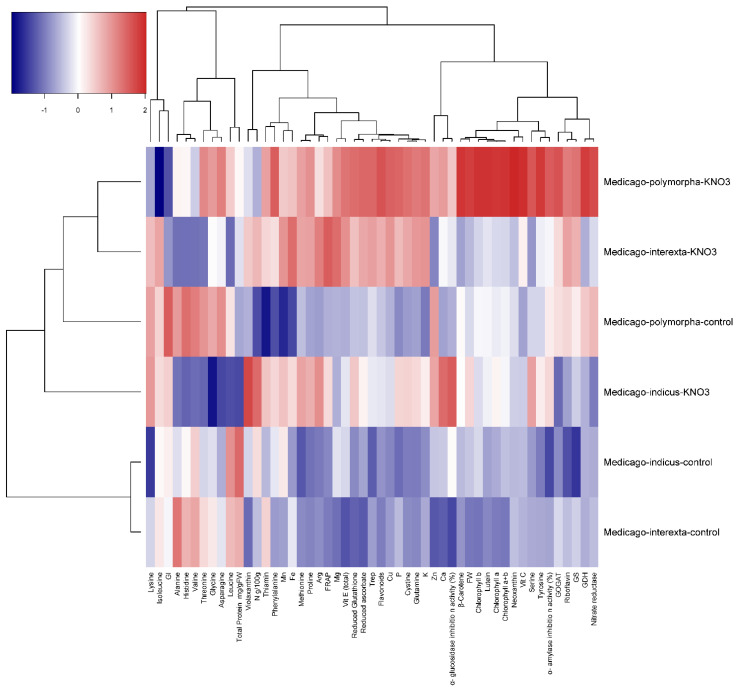
The cluster heatmap of primary and secondary metabolites of three *Medicago* species KNO_3_ priming treatment. The graph’s horizontal axis shows different treatments for each species, and the vertical axis shows different phytocompounds, amino acids, and nitrogen content. Color gradients represent the different values of contents under KNO_3_ priming compared with that of control.

**Figure 6:**
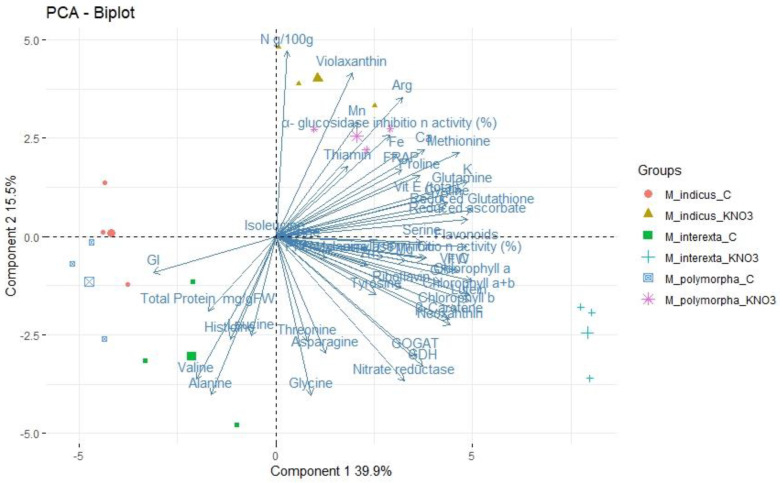
PCA-Biplot of *Medicago* species.

PCA is a multivariate statistical analysis that can be used to examine and simplify complex and huge datasets. The pattern of variation in *Medicago* species was also analyzed using principal component analysis (PCA) to evaluate the variety of the species and their link with the observed traits based on the correlation between the traits and extracted clusters. The chemical profiles of plants that were not primed with KNO_3_ were grouped and clearly separated from sprouts primed with KNO_3_. In our dataset, two groups of traits were identified in the PCA biplot considering both PC1 and PC2 simultaneously (Figure 5 and Figure 6). The FW, leaf pigments, mineral content, N metabolism, polyphenols, antioxidants enzymes, and glucosidase and amylase inhibitor enzymes linked with *Medicago interexta* and *polymorpha* primed with KNO_3_.

## 4. Discussion 

### 4.1. KNO_3_ Priming Increased Biomass Accumulation in Medicago Sprouts 

Seed priming is used to ensure rapid and uniform seed germination and seedling emergence in order to improve agricultural production performance. Priming with KNO_3_ has also been found to boost seedling germination, growth, establishment, and productivity in numerous studies. Seed priming has been extensively studied in terms of plant ecology, physiology, cellular biology, and molecular biology [28,29]. Increased seed quality has recently become a top focus in the agriculture business. Seed priming with KNO_3_ increased the growth of three *Medicago* species in the current study. The results were similar to those of earlier studies, which reported that KNO_3_ priming enhanced cucumber [30], white clover [31], and soybean [32] seedling fresh weights, when compared with unprimed seedlings. Similarly, seeds of *Medicago sativa* var. Anand-z primed with 0.1 percent MgCl_2_ also had a high rate of seed germination and seedling growth [33]. Enhancement of *Medicago* growth might be due to increased cell division and elongation and activation of ROS scavenging enzymes in KNO_3_ primed seeds [30]. KNO_3_ may promote the growth of *Medicago* sprouts by acting as nutrients and initiators of crucial emergence and growth processes in sprouts. The improvement of growth by KNO_3_ priming seemed to be varied among the species of *Medicago*. Indeed, *Medicago interexta* presented the highest biomass accumulation compared to other species. Differences in emergence and growth among species in response to germination conditions were reported in several research works [34]. The results could be confirmed by the photosynthetic pigments contents of *Medicago* sprouts, where the contents of chla, chlb, and total chlorophyll were generally significantly increased in response to KNO_3_ priming sprouts. Furthermore, the enhancement of chlorophyll biosynthesis by KNO_3_ was inconsistent with the increases of Mg^2+^ contents in *Medicago polymorpha* and *Medicago interexta*. The chlorophyll molecule containing Mg covalently linked with four nitrogen (N) atoms, and this might be the reason that KNO_3_ priming enhanced nutrients uptake and resulted to enhanced chlorophyll contents in *Medicago* sprouts leaves. The results are in agreement with previous studies, which reported that biopriming significantly enhanced chlorophyll contents in wheat leaves [35]. Furthermore, many works reported that lucerne possesses detoxifying and anticancerogenic properties due to its high chlorophyll content [35]; thus, it seemed that KNO_3_ priming could enhanced these proprieties in the three species. Moreover, the results revealed that the KNO_3_ priming enhanced the two groups of carotenoids, including *β-carotene* and hydroxylated carotenoids, designated as xanthophyll pigments, such as lutein, neoxanthin, and violaxanthin. Many reports revealed that *Medicago* contains 400–500 mg total carotenoids/kg, with the majority being xanthophylls, such as lutein and zeaxanthin [36]. Furthermore, a review of xanthophylls’ possible functions in disease prevention has revealed that they may have a preventive impact against some malignancies, coronary heart disease, and stroke. Besides, lutein, neoxanthin, and violaxanthin pigments possess anti-inflammatory and strong antioxidant properties, and they are very active against liver neoplasms [37].

### 4.2. KNO_3_ Priming Increases Nutritive Values of Medicago Sprouts

Mineral and vitamin deficiencies have been linked to a variety of detrimental health impacts in humans. Sprouts have long been thought to be a good source of bioavailable minerals, such as Fe, Zn, Mn, Mg, Cu, and Ca [38]. As a result, using KNO_3_ priming to increase the mineral element content of sprouts could improve the nutritional and health-promoting effects of *Medicago* sprouts. In the present study, KNO_3_ priming resulted in a significant increment in minerals nutrition concentration (Ca, Cu, Fe, Mn, K, and P) in the three species. The mineral content of sprouts is highly dependent on the sprouting conditions in general; however, the mineral contents observed in the sprouts analyzed in this study are consistent with those described in the literature [1]. Supporting our results, KNO_3_ priming enhanced leaf nutrient accumulation and significantly enhanced seedling growth in mung beans [29] and cucumber seedlings [30]. Furthermore, the increase in mineral content varied between species. *Medicago interexta* presented the highest increment in Ca content. The high content of Ca makes these products suitable for consumers with lactose intolerance [39]. Other minerals, such as Fe, were also quantified in *Medicago interexta*, being higher than the ones reported for other species. The high content of Fe could be helpful to prevent anemia caused by iron deficiency [39].

Concerning vitamins, this outcome revealed that the *Medicago* sprouts were rich in Vit E (tocopherol), and its content varied between species, since *Medicago polymorpha* and *Medicago interexta* showed the greatest levels. Besides, KNO_3_ priming enhanced the accumulation of Vit E and riboflavin in the three species, but it decreased the level of thiamine in *Medicago polymorpha*. The positive effect of KNO_3_ priming on vitamins content makes *Medicago* species a remarkable source of vitamins for supporting immunity.

### 4.3. KNO_3_ Priming Promotes N assimilation in Medicago Sprouts 

It is widely recognized that the essential amino acid content in plants is well acknowledged to have a substantial impact on their nutritional and health-promoting characteristics. Essential amino acids are critical for human health since they cannot be generated from scratch and act as building blocks for a variety of proteins that play critical roles in human health [40]. For instance, lucerne was approved for use in human nutrition [41]. The outcomes of the present study revealed that *Medicago* sprouts were rich in essential and semi-essential amino acids, such as glutamine, phenylalanine, threonine, asparagine, and glycine, which are considered as the most important bioactive components. Further, KNO_3_ priming enhanced the accumulation of proline, histidine, valine methionine cystine, and isoleucine. Moreover, the effect of KNO_3_ priming varied between the three species, since some amino acids greatly increased *Medicago polymorpha* and *Medicago interexta* more than *Medicago indicus.* In line with our findings, a previous study discovered that the response of plants to environmental conditions in primary N absorption differed by species [13]. The amino acid in *Medicago* sprouts is present in substantially larger concentrations than in eggs or wheat and was approved for use in human nutrition [41]. This increment in several amino acids in response to KNO_3_ priming has resulted in increases in total N in the three species and total protein in *Medicago interexta.* The increase in the activity of important enzymes in N metabolism, including NR, GS, GDH, and GOGAT, could explain the changes in amino acid levels. We suggested that the nitrate supplemented by the priming with KNO_3_ had a positive effect on NR. In fact, the increases in the activity of nitrate reductase (NR) increased the potential for nitrate reduction, resulting in increased capacity for amino acid synthesis, protein synthesis, and total N assimilation [12]. These results were in accordance with the results obtained in leaves of wheat seedlings [12] and Safflower [42] treated with KNO_3_. Concerning the total protein, the increase in this compound in response to KNO_3_ priming, except in *Medicago interexta*, may be due to the direct involvement of K in several steps of the translocation process, including the binding of RANt to ribosomes [13]. Interestingly, here, the KNO_3_ priming effect on N level and GS, GDH, and GOGAT enzymes activities were varied with different species.

### 4.4. Antioxidant Metabolites Accumulation Increased Antioxidant Biological Activity of Medicago Sprouts Extracts

The bioactive compounds of *Medicago* have gotten a lot of interest because of their antibacterial, anti-inflammatory, anticancer, and antioxidant properties [7]. These biological actions, which include anti-inflammatory and antioxidant properties, may be due to the presence of phenolic and flavonoids chemicals, which function as free radical scavengers and/or metal chelators [43]. Caunii et al. (2012) [44] found that the lucerne extract included a number of free hydroxyl groups (hydrogen donors), giving the product a significant antioxidizing effect in the human body. As such, the outcomes of the current study revealed that KNO_3_ enhanced the antioxidants properties of *Medicago* sprouts, and the increases depend on species. Indeed, *Medicago polymorpha* exhibit the highest antioxidant activities since flavonoids reduced glutathion and ascorbate content, and FRAP showed an increment by 2-fold, compared to control. This suggests that the obtained values of these compounds are impacted by species and germination processes.

As antidiabetic agents, the leaves of *Medicago* have been used traditionally to reduce plasma glucose levels in diabetic subjects [45,46]. In this investigation, the accumulation of nitrogen compounds enhanced by KNO_3_ priming increased the antidiabetic activity of *Medicago* sprouts extracts, since the GI decrease sharply mainly in *Medicago interexta* compared to others sprouts in response to KNO_3_ priming. Furthermore, the current study illustrated that KNO_3_ priming enhanced the inhibitor effect against α-amylase and α-glucosidase in the three species of *Medicago* sprouts. Following the previous studies and examination, the anti-diabetic activity of *Medicago* was tested against α-amylase [46]. The accumulation of N leads to an increase in amino acid synthesis. Hence, amino acids may regulate insulin secretion in several ways, including the production of metabolic coupling factors, plasma membrane depolarization, and mitochondrial function augmentation [40]. Furthermore, the enhancement in several enzymes of N metabolism caused a high production of glutamine. This former amino acid has been postulated to play a role in nutrient-induced stimulus-secretion coupling as an additive factor in the glucose-stimulated insulin secretion amplification pathway [40]. Overall, as antidiabetic agents, accumulation of nitrogenous compounds can explain the increased antidiabetic activity of *Medicago* sprouts extracts.

### 4.5. Species-Specific Responses to KNO_3_ Priming 

According to hierarchical clustering, the effect of KNO_3_ seemed to be related to *Medicago* species (Figure 3). The genetic diversity of *Medicago* has been identified by numerous studies [47]. Besides, *Medicago* was considered as the genetically complex species [48]. These findings indicate that there is a genotypic difference in seed priming efficacy, which is in accordance with other studies [47,48]. The differences between the three species may be due to ontogeny and species diversity. *Medicago indicus* sprouts primed by KNO_3_ showed the highest antioxidant and antidiabetics activities. Besides, it seemed that KNO_3_ improved the nutritive value of these farmer sprouts by enhancing the accumulation of vitamins (Vit E and Vit C), proteins, mineral nutrients, and nitrogen, followed by *Medicago interexta* and *Medicago polymorpha*, which responded to KNO_3_ priming by enhancing the accumulation of the major amino acids.

## 5. Conclusions

The use of KNO_3_ priming to improve the biological and nutritional qualities of *Medicago* sprouts has been proven to be effective. Hence, *Medicago* sprouts are increasingly being used as an alternative source of natural antioxidant and mineral components in ready-to-eat fresh products or the manufacture of new safe functional foods. At the species level, *Medicago interexta* and *Medicago polymorpha* were more responsive to the KNO_3_ positive effect, being better than other species (*Medicago indicus*), since it gave the highest antioxidant and antidiabetic activities.

## Figures and Tables

**Table 1:** Effect of KNO_3_ priming on nutriments content of three species of *Medicago* sprouts. Values are represented by mean ± standard deviation of at least three independent replicates. Means marked by different letters are significantly different than control at *p* < 0.05.

Parameters	Control			KNO_3_ Priming		
	*M. indicus*	*M. polymorpha*	*M. interexta*	*M. indicus*	*M. polymorpha*	*M. interexta*
Ca mg·g^−1^ dw	17.57 ± 2.35 b	12.68 ± 1.87 b	15.79 ± 3.5 b	27.79 ± 6.69 a	19.63 ± 3.48 a	25.17 ± 0.46 a
Cu μg·g^−1^ dw	2.26 ± 0.71 a	2.42 ± 0.79 a	2.57 ± 1.06 b	2.87 ± 0.28 a	3.963 ± 0.86 a	4.38 ± 1.13 a
Fe μg·g^−1^ dw	3.99 ± 0.238 b	4.80 ± 1.06 b	3.15 ± 0.78 b	5.48 ± 1.02 a	6.798 ± 1.69 a	5.76 ± 0.44 a
Zn μg·g^−1^ dw	22.62 ± 2.08 b	13.49 ± 1.10 b	36.61 ± 3.25 a	35.8 ± 3.21 a	17.24 ± 1.50 a	35.71 ± 3.16 a
Mn μg·g^−1^ dw	0.25 ± 0.12 a	0.19 ± 0.13 b	0.13 ± 0.13 b	0.27 ± 0.12 a	0.30 ± 0.12 a	0.27 ± 0.12 a
Mg mg·g^−1^ dw	2.90 ± 0.160 a	2.38 ± 0.10 b	2.69 ± 0.14 b	2.67 ± 0.12 a	3.81 ± 0.22 a	3.65 ± 0.22 a
K mg·g^−1^ dw	15.7 ± 1.311 b	20.8 ± 1.76 b	12.0 ± 0.98 b	40.6 ± 3.57 a	60.5 ± 5.50 a	67.3 ± 6.02 a
P mg·g^−1^ dw	5.8 ± 0.57 b	7.6 ± 0.595 b	6.5 ± 0.46 b	10.4 ± 0.84 a	11.2 ± 0.93 a	13.6 ± 1.13 a

**Table 2:** Effect of KNO_3_ priming on vitamin content of three species of *Medicago* sprouts. Values are represented by mean ± standard deviation of at least three independent replicates. Means marked by different letters are significantly different than control at *p* < 0.05.

Parameters	Control			KNO_3_ Priming		
	*M. indicus*	*M. polymorpha*	*M. interexta*	*M. indicus*	*M. polymorpha*	*M. interexta*
Vit C (mg·g^−1^ FW)	7.81 ± 1.35 b	7.67 ± 1.33 b	7.31 ± 1.26 b	8.15 ± 2.39 a	9.48 ± 1.37 a	13.92 ± 0.76 a
Vit E (mg·g^−1^ FW)	47.46 ± 1.17 a	38.05 ± 1.09 b	44.57 ± 1.58 b	48.47 ± 4.40 a	59.72 ± 2.43 a	61.92 ± 3.86 a
Thiamin (mg·g^−1^ FW)	0.10 ± 0.00 b	0.13 ± 0.02 a	0.06 ± 0.02 b	0.13 ± 0.02 a	0.13 ± 0.07 a	0.14 ± 0.06 a
Riboflavin (mg·g^−1^ FW)	0.30 ± 0.05 b	0.52 ± 0.09 b	0.75 ± 0.22 b	0.46 ± 0.22 a	0.87 ± 0.07 a	0.96 ± 0.46 a

**Table 3:** Effect of KNO_3_ priming on total amino acids content, total N, proteins and NR, GDH and GOGAT activities of three species of *Medicago* sprouts, Values are represented by mean ± standard deviation of at least three independent replicates, Means marked by different letters are significantly different than control at *p* < 0.05.

Parameters	Control			KNO_3_ Priming		
	*M. indicus*	*M. polymorpha*	*M. interexta*	*M. indicus*	*M. polymorpha*	*M. interexta*
**Amino acids (mg·g^−1^ fw)**						
Asparagine	1.64 ± 0.29 a	1.71 ± 0.38 b	1.9 ± 0.35 b	1.79 ± 1.52 a	1.99 ± 1.73 a	2.2 ± 1.93 a
Glutamine	1.86 ± 0.41 b	1.96 ± 0.45 b	2.17 ± 0.53 b	3.56 ± 3.32 a	4.07 ± 3.95 a	4.27 ± 4.23 a
Serine	1.37 ± 0.04 b	1.43 ± 0.15 a	1.59 ± 0.08 b	2.03 ± 2.16 a	1.25 ± 1.35 b	2.02 ± 2.48 a
Glycine	1.56 ± 0.28 a	1.68 ± 0.41 a	1.83 ± 0.28 b	1.18 ± 1.16 b	1.88 ± 1.62 a	2.02 ± 1.86 a
Arginine	0.31 ± 0.1 b	0.35 ± 0.15 b	0.37 ± 0.17 b	0.88 ± 0.78 a	0.84 ± 0.81 a	0.41 ± 0.61 a
Alanine	0.58 ± 0.1 a	0.71 ± 0.03 a	0.68 ± 0.12 a	0.51 ± 0.5 a	0.57 ± 0.5 a	0.65 ± 0.61 a
Proline	1.19 ± 0.44 b	1.34 ± 0.46 b	1.59 ± 0.59 b	2.67 ± 3.11 a	2.37 ± 3.26 a	3.09 ± 3.42 a
Histidine	0.74 ± 0.11 a	0.81 ± 0.11 a	0.89 ± 0.11 a	0.58 ± 0.58 b	0.41 ± 0.59 b	0.52 ± 0.74 b
Valine	0.89 ± 0.38 a	0.97 ± 0.43 a	1.05 ± 0.48 a	0.79 ± 0.54 a	0.65 ± 0.55 b	0.96 ± 0.71 b
Methionine	0.47 ± 0 b	0.55 ± 0 b	0.63 ± 0 b	0.85 ± 0.89 a	0.86 ± 0.89 a	0.86 ± 0.89 a
Cystine	0.87 ± 0.06 b	0.89 ± 0.03 b	0.92 ± 0.06 b	1.44 ± 1.17 a	1.45 ± 1.26 a	1.41 ± 1.37 a
Isoleucine	1.11 ± 0.72 b	1.12 ± 0.76 b	1.14 ± 0.8 b	1.39 ± 1.13 a	1.46 ± 1.17 a	1.28 ± 0.95 a
Leucine	1.04 ± 0.15 a	1.01 ± 0.29 a	0.98 ± 0.43 b	1.02 ± 0.86 a	1.15 ± 0.87 a	1.34 ± 1 a
Phenylalanine	1.98 ± 1.08 a	1.82 ± 1.17 a	1.65 ± 1.45 b	1.88 ± 2.14 a	1.13 ± 2.11 b	2.27 ± 2.44 a
Tyrosine	0.31 ± 0.08 b	0.32 ± 0.05 b	0.33 ± 0.05 b	0.4 ± 0.35 a	0.44 ± 0.34 a	0.47 ± 0.41 a
Lysine	0.77 ± 0.14 b	0.84 ± 0.1 b	0.92 ± 0.07 a	1.07 ± 0.92 a	1 ± 0.9 a	0.85 ± 0.82 b
Threonine	1.33 ± 0.28 a	1.39 ± 0.26 a	1.45 ± 0.24 a	1.47 ± 1.25 a	1.23 ± 1.26 a	1.59 ± 1.46 a
Tryptophan	0.78 ± 0.21 b	0.84 ± 0.23 b	0.9 ± 0.24 b	1.11 ± 0.92 a	1.32 ± 1.04 a	1.36 ± 1.1 a
**Nitrogen content and metabolism**						
Nitrogen (g/100 g)	23.39 ± 0.89 b	19.69 ± 0.82 b	15.72 ± 0.53 b	28.11 ± 1.24 a	24.95 ± 0.72 a	19.20 ± 0.8 a
Total protein (mg/g FW)	169.5 ± 1.9 a	153.61 ± 8.86 a	118.0 ± 3.16 b	99.6 ± 2.27 b	129.0 ± 8.47 b	136.8 ± 2.81 a
Nitrate reductase μmol nitrite/mg protein.min	45.24 ± 0.03 a	49.53 ± 2.47 b	86.19 ± 5.45 b	33.11 ± 2.23 b	56.36 ± 0.91 a	118 ± 11.27 a
GDH μmol NADH/mg protein.min	4.14 ± 0.21 a	4.10 ± 0.09 a	6.99 ± 0.48 b	4.14 ± 0.21 a	4.10 ± 0.09 a	10.33 ± 0.48 a
GOGAT μmol NADH/mg protein.min	7.83 ± 0.28 a	11.16 ± 0.45 b	14.35 ± 0.45 b	6.35 ± 0.29 b	14.70 ± 0.52 a	21.38 ± 1.83 a
GS μmol γ-glutamyl hydroxamate/mg protein.min	16.12 ± 0.91 b	23.09 ± 1.24 b	26.16 ± 0.45 b	23.00 ± 1.24 a	29.2 ± 0.77 a	32.55 ± 0.8 a
